# Early Diagnosis of COPD—How Can we Do Better?

**DOI:** 10.1002/resp.70155

**Published:** 2025-11-05

**Authors:** Shawn D. Aaron

**Affiliations:** ^1^ The Ottawa Hospital Research Institute University of Ottawa Ottawa Ontaria Canada

**Keywords:** COPD, diagnosis, epidemiology

Undiagnosed COPD is a major global health problem. Studies from across the world suggest that as many as 70% of adults with COPD remain undiagnosed [1]. A collaborative study assessed the prevalence of undiagnosed COPD in 27 countries and found COPD in 2995 of 30,874 adult participants (9.7%); of these 81.4% of cases were undiagnosed [2]. The myriad reasons for under‐diagnosis of COPD are highlighted in Table 1.

**TABLE 1:** Factors that may be associated with underdiagnosis of COPD.

Patient‐related factors	Healthcare provider factors	Healthcare system factors
Patients may under‐recognise and/or under‐report their symptoms.	Providers may have poor understanding of COPD diagnostic criteria.	Many healthcare systems lack convenient access to diagnostic spirometry.
Patient may limit their activities to minimise their breathlessness.	Providers may have inadequate training in the use and interpretation of spirometry.	Patients most likely to have COPD may belong to disadvantaged, lower socio‐economic groups, and disparities in access to health care for these groups, even in high‐income countries, can impact on diagnosis.
Some patients with undiagnosed COPD may have milder disease or milder impairment making diagnosis less likely.	Providers may not adequately investigate patients with respiratory symptoms.	Patients in low and middle‐income countries may have poorer access to quality healthcare resources.
	Providers may not refer patients with respiratory symptoms to specialists.	

Our current healthcare system generally fails many patients with COPD, since most remain undiagnosed until they develop moderate or severe airflow obstruction, and generally they present with significant disability, or an acute exacerbation, at the time of first diagnosis [3]. Consequently, most patients diagnosed with COPD in clinical practice are only recognised when their disease is already relatively advanced, and therapy is less effective. As has been shown for lung cancer detection in at‐risk subjects [4], screening or case‐finding are potential strategies to dramatically change the paradigm and identify patients earlier.

Early diagnosis of COPD can be potentially achieved by case‐finding. Case‐finding involves assessment of at‐risk individuals who present with unexplained respiratory symptoms. Case‐finding uses symptom questionnaires and may also make use of peak expiratory flow monitors or micro‐spirometers, to identify symptomatic individuals, or people at particularly high risk for COPD, who would benefit from diagnostic spirometry [5]. Case‐finding facilitates earlier identification of disease and can allow clinicians to direct non‐pharmacologic and pharmacologic treatments to these individuals.

How should we try to find individuals with undiagnosed COPD? One obvious approach would be to try to find them in primary care practices. There are several potential problems with this approach. Studies of patients with undiagnosed COPD and asthma suggest that many individuals with undiagnosed COPD tend to discount their symptoms, and they do not complain to their primary care practitioners about their respiratory symptoms [6]. Similarly, many individuals with undiagnosed COPD do not have family doctors, or they may see their family doctors very infrequently, and these individuals may be missed if case‐finding is confined to primary care practices [6].

A recent cluster‐randomised clinical trial tried to operationalise COPD case‐finding in primary care offices using the CAPTURE case‐finding tool [7]. Unfortunately, the study found that the use of the CAPTURE tool in primary care did not influence practitioners to order more spirometry, or make more diagnoses of COPD, compared to usual care. Furthermore, patients within the primary care practices randomised to the CAPTURE intervention did not report better health status, or experience fewer urgent visits for respiratory illness, compared to those within practices randomised to usual care. The investigators of the CAPTURE study pointed out that the results of the CAPTURE questionnaire were shared with clinical staff after the completion of the patient visit, and that the majority of patient visits were for reasons unrelated to respiratory illness [7]. In this context, it is not surprising that busy primary care practitioners therefore failed to act on the results of the CAPTURE questionnaire.

Another approach to find individuals with undiagnosed COPD is to find them within their homes and communities. The Undiagnosed COPD and Asthma in the Population (UCAP) Study was a multicenter, study that randomly dialed cellphones and landlines across Canada and telephone interviewed almost 27,000 adults with symptoms of respiratory disease using case‐finding questionnaires [8]. After exclusion of many people who had pre‐existing diagnosed lung disease, the investigators conducted pre and post‐BD spirometry in 2857 individuals who had no prior history of diagnosed lung disease. Of the 2857 individuals who underwent spirometry, 595 (21%) were found to have undiagnosed asthma or COPD. Over a one‐year follow‐up period, individuals with undiagnosed asthma or COPD who were randomised to guideline‐based care by a pulmonologist had less than half the rate of patient‐initiated healthcare utilisation events for respiratory illness, and significantly greater one‐year improvements in health‐related quality of life, symptoms, and lung function, compared to those randomised to usual care [8].

The UCAP study was the first to conduct case‐finding for COPD within the community and to couple early diagnosis to an intensive treatment intervention. While the UCAP study was successful, computer‐generated random digit dialing of all households was expensive and relatively inefficient. Cost for the random‐digit calls was > $450,000 Canadian, and more than one million random calls needed to be made to ultimately find 595 individuals with undiagnosed obstructive lung disease. The next step is to make COPD case‐finding within the community more feasible, and affordable, within our healthcare systems.

We are currently conducting a clinical trial of community‐based, patient‐initiated diagnosis of obstructive lung disease. Individuals experiencing unexplained respiratory symptoms complete a web‐based case‐finding questionnaire on‐line [9], and if their responses yield a risk score exceeding a specified threshold, they are referred via a web‐based program for diagnostic spirometry. We are advertising the web‐based case‐finding questionnaire locally within communities. Information is being posted in local community centers, and in community‐based newsletters and local community newspapers, including those targeting ethnic groups and language and cultural minorities. Finally, we are also using local radio advertisements to reach broadly within communities.

Achieving earlier diagnosis of COPD, via a patient‐initiated community‐based case‐finding strategy, will ensure that symptomatic patients are not left undiagnosed and untreated. Ultimately this approach will help patients and may provide health economic benefits to society and to our healthcare systems.

## Conflicts of Interest

The author declares no conflicts of interest.
